# Effect of a Psychomotor Intervention on Motor Competence, Graphomotor Performance and Writing Proficiency in 5- to 6-Year-Old Preschool Children

**DOI:** 10.3390/children13070973

**Published:** 2026-07-22

**Authors:** Nerea Blanco-Martínez, Daniel González-Devesa, Eva María Doval-Garabatos, Carlos Ayán-Pérez

**Affiliations:** 1 Departamento de Didácticas Especiáis, Universidade de Vigo, 36310 Vigo, Spain; n.blanco@uvigo.gal (N.B.-M.); cayan@uvigo.es (C.A.-P.); 2 Well-Move Research Group, Galicia Sur Health Research Institute (IIS Galicia Sur), SERGAS-UVIGO, 36310 Vigo, Spain; 3 Grupo de Investigación en Actividad Física, Educación, y Salud (GIAFES), Universidad Católica de Ávila, C/Canteros, 05005 Avila, Spain; 4 Facultade de Ciencias da Educación e do Deporte de Pontevedra, Universidade de Vigo, 36310 Vigo, Spain; dovaal.eva@gmail.com

**Keywords:** psychomotor performance, handwriting, motor skills, child, early intervention

## Abstract

**Highlights:**

**What are the main findings?**

• A six-week psychomotor intervention was associated with significantly greater pre–post improvements in the motor competence tasks assessed, including manual dexterity, aiming, and catching.
• The intervention was associated with significantly greater pre–post improvements in graphomotor performance, while pencil grip was the only writing proficiency item showing a significant between-group difference at post-intervention.

**What is the implication of the main finding?**

• Psychomotor activities may be a feasible school-based strategy to support motor competence and graphomotor development in preschool children.
• Larger and longer studies are needed to determine whether motor gains translate into broader and sustained improvements in writing proficiency.

**Abstract:**

**Background/Objectives**: Early handwriting and motor skills underpin later academic success, yet robust evidence for psychomotor programs in preschoolers is limited. This study examined whether participation in a six-week psychomotor intervention was associated with changes in graphomotor performance, writing proficiency, and motor competence in preschool children. **Methods**: This non-randomized quasi-experimental study included pre- and post-intervention assessments and a control group. Thirty-four preschoolers were assigned by class to an intervention group (*n* = 19) or a control group (n = 15). For six weeks, the intervention group completed three 50 min psychomotor sessions per week targeting fine- and gross-motor abilities, whereas the control group continued its usual school routine. Graphomotor performance (Pascual Graphomotor Test), writing proficiency (seven-item teacher scale) and motor competence (Movement Assessment Battery for Children-2) were assessed one week before and after the program. **Results**: The intervention group showed significant improvements in graphomotor performance and all motor-skill tasks assessed. Between-group comparisons of pre–post change favored the intervention group for the total graphomotor score (mean difference = −3.14 points, 95% CI [−4.59, −1.68]; *p* < 0.001; Hedges’ g = −1.48) and for all four motor-skill tasks (*p* ≤ 0.019). At post-intervention, pencil grip was the only writing proficiency item showing a significant between-group difference (*p* = 0.002; Cramér’s V = 0.617), whereas no significant differences were observed for the remaining items. **Conclusions**: A psychomotor intervention was associated with greater improvements in motor competence and graphomotor performance compared with the control condition. However, evidence for broader improvements in writing proficiency, including handwriting legibility, was limited. Although pencil grip differed between groups at post-intervention, this finding should be interpreted cautiously because the groups already differed at baseline.

## 1. Introduction

Handwriting represents a foundational skill in early childhood, with longitudinal research demonstrating significant associations between early handwriting proficiency and later academic outcomes [1]. Despite a notable decline in formal handwriting instruction over recent decades, converging evidence underscores its essential role in the development of fine motor control, visuomotor integration, and higher-order cognitive and linguistic functions [1,2]. The act of handwriting engages distributed neural networks involving motor, perceptual, and language systems, contributing not only to the acquisition of orthographic and written language knowledge but also to increased classroom engagement and broader academic success [3]. Accordingly, handwriting instruction may serve as a valuable intervention target for enhancing foundational academic competencies.

Although handwriting-related skills begin to emerge prior to formal schooling [4], early forms of writing behaviors, such as exploratory mark-making, are typically observed by the age of two. Around age three, children generally begin to acquire more structured cognitive, perceptual–motor, and linguistic components associated with handwriting [5]. This developmental trajectory extends throughout the preschool years, with many children attaining sufficient readiness for formal handwriting instruction by approximately five years of age [6]. However, handwriting readiness is not determined solely by chronological age; it also depends on the progressive development of fine motor coordination, in-hand manipulation, and visual–motor integration, which contribute to children’s ability to control a writing instrument and reproduce forms accurately [7,8]. Consequently, considerable interindividual variability may be observed among preschool children of similar ages.

Handwriting improvement strategies have primarily focused on instructional approaches such as motor imitation, visual cues, memory retrieval [9], or cognitive training [10]. Although task-specific practice remains a central component of handwriting learning, intervention programs may also address the underlying visual–motor and fine motor abilities that support successful written production [7,8]. However, scientific evidence remains limited, and further research is needed to evaluate the effectiveness of alternative interventions, particularly among preschool-aged children [11]. Within this framework, psychomotor interventions designed to improve perceptual and motor skills constitute a promising domain warranting further empirical investigation [12].

Handwriting itself constitutes a highly complex perceptual–motor skill, requiring the integration of visuomotor coordination, motor planning, cognitive processing, and tactile-kinesthetic perception [13]. The development of graphomotor skills—considered a subset of fine motor abilities—thus depends on the coordinated functioning of musculoskeletal and cognitive systems to enable the fluent and accurate production of written forms [14]. Although the development of these skills during the preschool years remains relatively underexplored [15], emerging evidence suggests their pivotal role in supporting later academic achievement and active participation in school-based tasks [16].

While existing evidence suggests that psychomotor approaches may enhance handwriting proficiency in preschool-aged children [17,18], findings across studies remain inconsistent, with some interventions failing to demonstrate significant effects [19]. Although interventions targeting foundational motor and perceptual skills—such as fine motor coordination, visual–motor integration, and bilateral coordination—have shown promising results in some studies, the overall body of research remains inconclusive, highlighting the need for further rigorous investigation [11].

Given the current evidence, this study aimed to examine whether participation in a six-week psychomotor intervention was associated with changes in graphomotor performance, writing proficiency, and motor competence in 5- to 6-year-old preschool children, compared with a control group continuing its usual school routine.

## 2. Materials and Methods

### 2.1. Participants

This non-randomized quasi-experimental intervention study, which included pre- and post-intervention assessments and a control group, involved healthy Spanish preschool children recruited from a kindergarten located in a rural area of northern Spain.

Inclusion criteria were: (a) age between 5 and 6 years and (b) absence of any medical condition that could interfere with the performance of field-based assessments. Children with intellectual or physical disabilities that impeded comprehension of the test procedures or accurate execution of the tasks were excluded. Written informed consent was obtained from the parents or legal guardians of all participants. The study protocol was approved by the Ethics Committee.

### 2.2. Measurements

#### 2.2.1. Graphomotor Performance

Graphomotor performance was assessed using the Pascual Graphomotor Test [20]. Participants were instructed to copy eight geometric figures (e.g., diamond, cross, flower). Errors were categorized as either graphic errors (scored as 1 point) or distortions (scored as 2 points), with a maximum possible total score of 20 points. Lower total scores reflected better graphomotor performance.

#### 2.2.2. Writing Proficiency

An exploratory observational scale was developed by the authors, in collaboration with an early childhood educator from the study site, to evaluate writing proficiency. The scale included seven items evaluating specific aspects of writing proficiency: pencil grip, deviation from lines, rightward slant, leftward slant, upright writing, letter size uniformity, and reversal of the letters s, c, and n. The seven items were selected based on the teacher’s pedagogical understanding of the developmental process of writing acquisition among the participating children. No formal operational definitions or written standardized scoring instructions were developed for the individual items; instead, their interpretation and scoring criteria were agreed upon jointly by the classroom teacher and the researcher responsible for the intervention. Items were scored on a 5-point Likert scale (never = 0 to always = 4), with lower scores indicating better writing proficiency. The scale was not formally piloted before data collection. Inter-rater and intra-rater reliability were not formally examined.

#### 2.2.3. Motor Competence

Selected tasks from the 4–6-year age band of the Movement Assessment Battery for Children-2 (MABC-2) were administered to evaluate participants’ motor competence. The MABC-2 is a standardized instrument designed to assess motor competence in children [21]. The selected tasks comprised Posting Coins and Turning Pegs, which assess manual dexterity, and Throwing a Beanbag onto a Mat and Catching with Two Hands, which assess aiming and catching abilities.

All assessments were conducted one week before and one week after the intervention. Graphomotor performance and writing proficiency were evaluated by the same classroom teacher for both groups at both assessment time points, while the MABC-2 was administered by a researcher who had received prior training in its administration. No blinding procedures were implemented in this study.

### 2.3. Procedure

The study was conducted at the kindergarten during school hours. Participants belonged to two pre-existing classes, and allocation was performed at the class level. Based on organizational feasibility, the research team assigned Class 6 “A” to the intervention group and Class 6 “B” to the control group.

All assessments were conducted one week before and one week after the intervention. The intervention was carried out during school hours, at approximately 12:30 p.m., after the children returned from recess and had completed their snack.

During the study period, both groups continued their usual school routines, including the motor and psychomotor activities normally provided by the kindergarten. In addition, the intervention group participated in the psychomotor program, whereas the control group did not receive this additional intervention.

Individual randomization was therefore not considered feasible within the school context. No a priori sample size calculation was performed because the available sample was determined by the number of eligible children enrolled in the two participating classes.

### 2.4. Intervention Description

The intervention lasted six weeks and comprised 18 sessions, delivered three times per week, with each session lasting approximately 50 min. The program was designed and delivered by EMDG, who had relevant training in the field. The classroom teacher did not participate in the design of the intervention.

The intervention combined table-top manipulative and hand–eye coordination activities with whole-body psychomotor activities conducted in the school psychomotor room. The program was organized around three recurring content blocks: (1) fine motor manipulation and manual dexterity; (2) visual–motor coordination and graphomotor-related abilities; and (3) global psychomotor skills, postural control, and motor planning. These blocks were not implemented as separate consecutive phases but were alternated throughout the six-week intervention. No predetermined linear progression of difficulty was established. Instead, the different activity types and materials were revisited cyclically throughout the intervention.

Fine motor activities included tasks using purpose-designed materials, such as inserting figures through corresponding slots, threading a cord through numbered templates, and reproducing figures using colored sticks. These activities involved precise object manipulation, finger control and dissociation, regulation of grip force, spatial orientation, and coordination between visual information and hand movements. The program also included psychomotor circuits, passing games, and jumping activities, which involved dynamic balance, postural stability, whole-body coordination, and the planning and sequencing of movements.

The intervention did not include direct handwriting instruction. Instead, the activities were intended to stimulate motor abilities considered relevant to graphomotor performance. Fine motor manipulation was used to promote hand and finger control; visually guided tasks required the integration of visual information with precise manual actions; seated and whole-body activities involved postural stabilization; and both manipulative tasks and psychomotor circuits required children to organize, anticipate, and sequence their movements.

### 2.5. Statistical Analysis

All statistical analyses were performed using SPSS 15.0 (SPSS Inc., Chicago, IL, USA). The Shapiro–Wilk test was performed to check for normality of distribution. The assumption of homogeneity of variance was verified with Levene’s test. Data are presented as mean ± standard deviation (SD) for normally distributed variables, and median (interquartile range, IQR) if they did not show a normal distribution. Between-group comparisons were performed with an independent-samples *t*-test; if the assumptions of normality or homoscedasticity were violated, the non-parametric Mann–Whitney U test was used instead. Within-group comparisons were analyzed with a paired-samples *t*-test or, when appropriate, the Wilcoxon signed-rank test. Pre–post change scores were also calculated and compared between groups using the corresponding independent-samples test. Effect sizes were reported as Hedges’ g for between-group comparisons. Mean differences and 95% confidence intervals (CIs) were reported where appropriate. Statistical significance was set at *p* < 0.05.

Contingency tables were calculated to detect systematic associations of the assessed variables. Categorical variables were analyzed using a chi-square test of independence (α = 0.05). Adjusted standardized residuals (CR) were applied to isolate sources of variation among groups [22]. Cramér’s *V* was calculated as a measure of effect size for categorical comparisons.

## 3. Results

A total of thirty-four schoolchildren completed the study (age: 65 months; height: 1.12 ± 0.04 m; weight: 21.94 ± 2.40 kg), divided into an intervention group (n = 19) and a control group (n = 15). Sex distribution was similar between groups (boys: 63% vs. 67%; *p* = 0.832). The intervention group was slightly older (66 [3] vs. 64 [2] months) than the control group, and this difference reached statistical significance (*p* = 0.027). Table 1 shows baseline results for writing proficiency. Prior to the intervention, the groups differed in “pencil grip” (*p* < 0.005; Cramér’s *V* = 0.617) and “deviation from lines” (*p* = 0.043; Cramér’s *V* = 0.490). No significant between-group differences were found for the remaining variables.

Following the intervention, the total graphomotor score decreased significantly in the intervention group (mean change = −2.74 points, 95% CI [−3.94, −1.54]; *p* < 0.001), indicating an improvement in graphomotor performance, while the control group’s scores remained stable (mean change = 0.40 points, 95% CI [−0.35, 1.15]; *p* = 0.271). The post-intervention comparison between the two groups did not yield a statistically significant difference (mean difference = −0.86 points, 95% CI [−3.14, 1.41]; *p* = 0.446; Hedges’ g = −0.26). However, the pre–post change differed significantly between groups, with a greater reduction in the intervention group than in the control group (mean difference = −3.14 points, 95% CI [−4.59, −1.68]; *p* < 0.001; Hedges’ g = −1.48) (Table 2).

Regarding writing proficiency outcomes (Table 3), the only item showing significant differences between groups was “pencil grip” (*p* = 0.002; Cramér’s *V* = 0.617), with the intervention group displaying a higher percentage of consistently appropriate grip (“always”) (63%) compared to the control group (33%). No significant differences were observed in the remaining writing proficiency items (*p* > 0.09).

Pre–post comparisons showed statistically significant improvements in the intervention group across all motor competence domains (Table 2). In contrast, the control group showed a significant change only in the Turning Pegs task, with performance slightly declining (mean change = 0.58 s, 95% CI [0.07, 1.09]; *p* = 0.027). Post-intervention comparisons revealed significant differences between groups across all motor-skill tasks. Moreover, pre–post change differed significantly between groups for all four motor-skill tasks: Posting Coins (mean difference in change = −3.41 s, 95% CI [−5.19, −1.64]; *p* < 0.001; Hedges’ g = −1.32), Turning Pegs (mean difference in change = −2.25 s, 95% CI [−3.35, −1.16]; *p* < 0.001; Hedges’ g = −1.41), Throwing a Beanbag (*p* = 0.008; rank-biserial r = 0.53), and Catching with Two Hands (*p* = 0.019; rank-biserial r = 0.45), consistently favoring the intervention group (Table 2).

## 4. Discussion and Implications

The aim of this study was to examine whether a six-week psychomotor intervention was associated with changes in motor competence, graphomotor performance, and writing proficiency in preschool children. The results provide preliminary information for preschool teachers and other disciplines such as occupational therapy and sport science, as they suggest that psychomotor strategies may support the development of basic motor skills and fine motor control in the early stages of education.

The Pascual Graphomotor Test assesses graphomotor performance through tasks involving visuospatial and visuoconstructive functions [23]. In this study, the total graphomotor score decreased significantly within the intervention group, indicating improved graphomotor performance. The pre–post reduction was significantly greater in the intervention than in the control group. However, the groups did not differ significantly in their absolute post-intervention scores. Therefore, the findings indicate a greater pre–post improvement in graphomotor performance in the intervention group, despite the absence of a significant between-group difference in absolute post-intervention scores. The lack of a significant post-intervention difference may be related to the small sample size or the limited duration of the intervention. Moreover, although improvements in fine motor skills support graphomotor abilities [17], this alone may not necessarily translate into broader improvements in writing proficiency or handwriting legibility.

Pencil grip refers to the position adopted by the fingers in relation to the pencil [24], while the alignment of words on the line is a basic feature of functional writing [25]. Regarding individual writing proficiency variables, pencil grip was the only item showing a significant between-group difference after the intervention, favoring the intervention group. However, because pencil grip already differed significantly between groups at baseline, this post-intervention difference should not be interpreted as evidence of an improvement attributable to the intervention. Deviation from lines also differed significantly between groups at baseline but did not show a significant between-group difference at post-intervention. Therefore, these item-level findings, derived from an exploratory and non-validated observational scale, should be interpreted cautiously and provide only preliminary evidence of broader changes in writing proficiency. Nevertheless, previous research has highlighted the relevance of intrinsic factors, such as fine motor control, bilateral and visuomotor integration, motor planning, and in-hand manipulation, as well as extrinsic factors, such as sitting posture and chair height, for writing proficiency and functional handwriting [26,27].

For the remaining writing proficiency variables, no significant post-intervention differences were found between groups in legibility-related aspects, such as letter slant, size uniformity, or reversal of specific letters. Although the intervention group showed a significantly greater pre-post improvement in overall graphomotor performance, this improvement was not accompanied by significant post-intervention differences in most items assessed using the writing proficiency exploratory teacher-rated scale. Previous research has reported improvements in writing proficiency outcomes, such as legibility and writing speed, following visuoperceptual–motor activities [19,26]. This lack of significant differences may be attributed to the short duration of the intervention, which may have been insufficient to produce measurable changes. Studies with a greater number of sessions in preschoolers, focused on games and motor activities that develop body awareness, cognition, sensory processing, and visuoperceptual skills, have improved both fine motor skills and pre-writing abilities [28]. Nonetheless, it is important to note that other interventions based on intensive handwriting practice have been considered more effective than visuoperceptual–motor training for improving legibility [19].

Competence in motor skills during preschool is essential not only for handwriting [18] but also for the child’s overall development [29]. At the level of motor competence, pre-post comparisons showed that the intervention group achieved significant within-group improvements in all motor skill tasks assessed using MABC-2, whereas the control group showed a significant decline in the turning pegs task and no significant changes in the remaining tasks. Furthermore, post-intervention comparisons favored the intervention group across all four motor-skill tasks, and the pre–post changes also differed significantly between groups for all four tasks, consistently favoring the intervention group. These findings are consistent with those of Hestbaek et al. [30] in Danish preschool children, where a motor skill-focused intervention produced positive short-term improvements, also assessed using the MABC-2. These findings suggest that the psychomotor intervention was associated with improvements in motor competence. However, although these motor gains were accompanied by a greater pre–post improvement in graphomotor performance, they were not accompanied by broader post-intervention differences in writing proficiency.

### Limitations

The main strength of this research lies in its original approach, as it is among the few studies to evaluate improvements in graphomotor performance and writing proficiency following a motor-based intervention. However, several limitations should also be acknowledged.

First, the sample size may have limited the ability to detect differences between groups, particularly in aspects related to writing proficiency. Second, baseline comparability between groups was limited, as significant pre-intervention differences were observed in age, pencil grip, and deviation from lines. These imbalances may have influenced post-intervention comparisons, particularly because children’s motor and graphomotor development are closely age-related [31]. Third, participants were allocated according to two pre-existing classes, with one class assigned to each condition by the research team based on organizational feasibility rather than randomization. As each condition was represented by a single class, the observed between-group differences may also reflect class-specific factors, such as classroom context, teaching practices, peer dynamics, or previous learning experiences. Consequently, these differences cannot be attributed exclusively to the intervention. Fourth, writing proficiency was assessed using an exploratory observational scale developed specifically for this study that had not been previously validated. No formal operational definitions or standardized scoring instructions were developed, the scale was not formally piloted, and its intra-rater and inter-rater reliability was not assessed. Therefore, findings derived from this measure should be interpreted with caution. Finally, the assessors were not blinded to group allocation or assessment time point, which may have introduced assessment bias, particularly given the small sample size.

## 5. Conclusions

The findings of this study suggest that the psychomotor intervention was associated with greater improvements in motor competence and graphomotor performance in preschool children. However, evidence for broader improvements in writing proficiency and legibility was limited, as no significant between-group differences were observed in the remaining writing proficiency variables. Although pencil grip was the only writing proficiency item showing a significant between-group difference at post-intervention, this finding cannot be attributed exclusively to the intervention because the groups already differed in this variable at baseline. Future studies with larger samples, random allocation, follow-up assessments, and validated writing proficiency instruments are needed to confirm the robustness and durability of the observed associations between psychomotor interventions and improvements in graphomotor performance and writing proficiency.

## Figures and Tables

**Table 1:** Writing proficiency scores at pre-intervention in the intervention (n = 19) and control groups (n = 15).

	Never	Very Few Times	Few Times	Many Times	Always	*p*-Value	*Cramér’s* *V*
**Pencil grip**						**<0.005**	**0.617**
Intervention group	21.1 (4) [1.9]	-	5.3 (1) [0.9]	10.5 (2) [−3.4]	63.2 (12) [1.7]		
Control group	0 (0) [−1.9]	-	0 (0) [−0.9]	66.7 (10) [3.4]	33.3 (5) [−1.7]		
**Deviation from lines**						**0.043**	**0.490**
Intervention group	47.4 (9) [0.4]	21.1 (4) [−2.3]	5.3 (1) [0.9]	26.3 (5) [2.2]	-		
Control group	40 (6) [−0.4]	60 (9) [2.3]	0 (0) [−0.9]	0 (0) [−2.2]	-		
**Rightward slant**						0.471	0.210
Intervention group	-	-	5.3 (1) [0.9]	57.9 (11) [0.7]	36.8 (7) [−1.0]		
Control group	-	-	0 (0) [−0.9]	46.7 (7) [−0.7]	53.3 (8) [1.0]		
**Leftward slant**						0.273	0.276
Intervention group	84.2 (16) [−1.6]	5.3 (1) [0.9]	10.5 (2) [1.3]	-	-		
Control group	100 (15) [1.6]	0 (0) [−0.9]	0 (0) [−1.3]	-	-		
**Upright writing**						0.216	0.300
Intervention group	-	-	5.3 (1) [0.9]	68.4 (13) [1.3]	26.3 (5) [−1.6]		
Control group	-	-	0 (0) [−0.9]	46.7 (7) [−1.3]	53.3 (8) [1.6]		
**Letter size uniformity**						0.497	0.203
Intervention group	-	-	21.1 (4) [1.2]	57.9 (11) [−0.5]	21.1 (4) [−0.4]		
Control group	-	-	6.7 (1) [−1.2]	66.7 (10) [0.5]	26.7 (4) [0.4]		
**Reversal of the letters s, c, and n**						0.393	0.234
Intervention group	84.2 (16) [−0.2]	15.8 (3) [0.8]	-	0 (0) [−1.1]	-		
Control group	86.7 (13) [0.2]	6.7 (1) [−0.8]	-	6.7 (1) [1.1]	-		

Data are presented as % (n) [Adjusted standardized residuals]; Bold values denote statistical significance (*p ˂* 0.05) among groups; Significant corrected typified residuals (−1.96 > rz > 1.96).

**Table 2:** Intra-group and inter-group results in motor competence and graphomotor performance.

	Intervention Group (n = 19)		Control Group (n = 15)		Total (N = 34)		*p*-Value Between-Group Post-Intervention
	Pre-Intervention	Post-Intervention	Pre-Intervention	Post-Intervention	Pre-Intervention	Post-Intervention	
**Motor Competence (MABC-2)**							
Posting Coins	21.68 ± 2.83	19.16 ± 2.63 *	21.52 ± 3.24	22.42 ± 2.80	21.61 ± 2.97	20.60 ± 3.13 ^§^	0.001
Turning Pegs	18.29 ± 2.40	16.62 ± 1.74 *	18.19 ± 2.01	18.77 ± 2.05 ^‡^	18.25 ± 2.21	17.57 ± 2.14 ^§^	0.002
Throwing a Beanbag	6 [3]	8 [1] *	5 [4]	6 [3]	6 [4]	7 [2.25] ^§^	<0.001
Catching with Two Hands	8 [1]	9 [1] ^‡^	8 [5]	8 [3]	8 [2]	9 [1.25] ^§^	<0.001
**Graphomotor performance**							
Total Score	14.47 ± 2.80	11.74 ± 3.28 *	12.20 ± 3.93	12.60 ± 3.18	13.47 ± 3.48	12.12 ± 3.20 ^§^	0.446

Mean ± standard deviation for normally distributed continuous variables, and median [interquartile range] for continuous variables that do not follow a normal distribution. * denotes significant within-group pre–post difference (*p* ≤ 0.01). ^‡^ denotes significant within-group pre–post difference (*p* ≤ 0.05). ^§^ denotes a significant between-group difference in pre–post change (*p* ≤ 0.05).

**Table 3:** Writing proficiency scores at post-intervention in intervention (n = 19) and control groups (n = 15).

	Never	Very Few Times	Few Times	Many Times	Always	*p*-Value	*Cramér’s V*
**Pencil grip**						**0.002**	**0.617**
Intervention group	-	-	26.3 (5) [2.2]	10.5 (2) [−3.4]	63.2 (12) [1.7]		
Control group	-	-	0 (0) [−2.2]	66.7 (10) [3.4]	33.3 (5) [−1.7]		
**Deviation from lines**						0.091	0.376
Intervention group	47.4 (9) [0.4]	31.6 (6) [−1.7]	21.1 (4) [1.9]	-	-		
Control group	40 (6) [−0.4]	60 (9) [1.7]	0 (0) [−1.9]	-	-		
**Rightward slant**						0.177	0.381
Intervention group	-	0 (0) [−1.1]	21.1 (4) [1.9]	31.6 (6) [−0.9]	47.4 (9) [0]		
Control group	-	6.7 (1) [1.1]	0 (0) [−1.9]	46.7 (7) [0.9]	46.7 (7) [0]		
**Leftward slant**						-	-
Intervention group	100 (19) [−]	-	-	-	-		
Control group	100 (15) [−]	-	-	-	-		
**Upright writing**						0.177	0.381
Intervention group	-	0 (0) [−1.1]	21.1 (4) [1.9]	31.6 (6) [−0.9]	47.4 (9) [0]		
Control group	-	6.7 (1) [1.1]	0 (0) [−1.9]	46.7 (7) [0.9]	46.7 (7) [0]		
**Letter size uniformity**						0.646	0.160
Intervention group	-	-	5.3 (1) [−0.2]	52.6 (10) [−0.8]	42.1 (8) [0.9]		
Control group	-	-	6.7 (1) [0.2]	66.7 (10) [0.8]	26.7 (4) [−0.9]		
**Reversal of the letters s, c, and n**						0.841	0.034
Intervention group	84.2 (16) [−0.2]	15.8 (3) [0.2]	-	-	-		
Control group	86.7 (13) [0.2]	13.3 (1) [−0.2]	-	-	-		

Data are presented as % (n) [Adjusted standardized residuals]; Bold values denote statistical significance (*p ˂* 0.05) among groups; Significant corrected typified residuals (−1.96 > rz > 1.96).

## Data Availability

The raw data supporting the results of this article will be made available by the authors on request, due to privacy considerations.
